# *Burkholderia dabaoshanensis* sp. nov., a Heavy-Metal-Tolerant Bacteria Isolated from Dabaoshan Mining Area Soil in China

**DOI:** 10.1371/journal.pone.0050225

**Published:** 2012-12-04

**Authors:** Honghui Zhu, Jianhua Guo, Meibiao Chen, Guangda Feng, Qing Yao

**Affiliations:** 1 Guangdong Provincial Microbial Culture Collection and Application Key Laboratory, Guangdong Open Laboratory of Applied Microbiology, State Key Laboratory of Applied Microbiology (Ministry–Guangdong Province Jointly Breeding Base ), South China, Guangdong Institute of Microbiology, Guangzhou, Guangdong, People’s Republic of China; 2 South China Agricultural University, Guangzhou, Guangdong, People’s Republic of China; Dowling College, United States of America

## Abstract

Heavy-metal-tolerant bacteria, GIMN1.004^T^, was isolated from mine soils of Dabaoshan in South China, which were acidic (pH 2–4) and polluted with heavy metals. The isolation was Gram-negative, aerobic, non-spore-forming, and rod-shaped bacteria having a cellular width of 0.5−0.6 µm and a length of 1.3−1.8 µm. They showed a normal growth pattern at pH 4.0–9.0 in a temperature ranging from 5°C to 40°C.The organism contained ubiquinone Q-8 as the predominant isoprenoid quinine, and C_16∶0_, summed feature 8 (C_18∶1_ *ω7c* and C_18∶1_ *ω6c*), C_18∶0_, summed feature 3 (C_16∶1_ *ω7c* or iso-C_15∶0_ 2-OH), C_17∶0_ cyclo, C_18∶1_ *ω9c*, C_19∶0_ cyclo *ω8c*, C_14∶0_ as major fatty acid. These profiles were similar to those reported for *Burkholderia* species. The DNA G+C % of this strain was 61.6%. Based on the similarity to 16S rRNA gene sequence, GIMN1.004^T^ was considered to be in the genus *Burkholderia*. The similarities of 16S rRNA gene sequence between strain GIMN1.004^T^ and members of the genus *Burkholderia* were 96−99.4%, indicating that this novel strain was phylogenetically related to members of that genus. The novel strain showed the highest sequence similarities to *Burkholderia soli* DSM 18235^T^ (99.4%); Levels of DNA-DNA hybridization with DSM 18235^T^ was 25%. Physiological and biochemical tests including cell wall composition analysis, differentiated phenotype of this strain from that closely related *Burkholderia* species. The isolation had great tolerance to cadmium with MIC of 22 mmol/L, and adsorbability of 144.94 mg/g cadmium,and it was found to exhibit antibiotic resistance characteristics. The adsorptive mechanism of GIMN1.004^T^ for cadmium depended on the action of the amide,carboxy and phosphate of cell surface and producing low-molecular-weight (LMW ) organic acids to complex or chelated Cd^2+^.Therefore, the strain GIMN1.004^T^ represented a new cadmium resistance species, which was tentatively named as *Burkholderia dabaoshanensis* sp. nov. The strain type is GIMN1.004^T^ ( = CCTCC M 209109^T^ =  NRRL B-59553^T^ ).

## Introduction

Activities such as mineral excavation, or transportation, smelting, refining, disposal of the tailings and waste waters around mines are important causes of heavy metals contamination [1], [2]. Heavy metals contamination on agricultural soils and crops in the vicinity of mine areas has been regarded as one of the most severe hazards to environmentaland human health [3], [4]. Treatment of heavy metals contaminated soil was urgent. Conventional methods including chemical precipitation, ion exchange or reverse osmosis processes were used to remove heavy metals from polluted soils, but there kinds of treatments were costly and showed several disadvantages, such as high reagent requirements and the generation of toxic sludge [5]. Compared with conventional methods, the bioremdation process using microbial biomass offers advantages of low costs, reagent requirement and minimization of the volume of sludge to be disposed [6]. Therefore, bioremediation using heavy-tolerant microorganism is an alternative method to remove or recover heavy metals efficiently from polluted environment, and isolation of heavy-metal-tolerant microbes as bioremediation agent is fundamentally important. *Burkholderia* species is an ubiquitous, microbe that are highly resistant to heavy metals (HMs). Many novel species of the genus Burkholderia have been described in recent years, certain species of Burkholderia have proved to be highly efficient in biocontrol, bioremediation [7], [8] and several mechanisms of heavy metal resistance are known, such as the formation and sequestration of heavy metals in complexes, reduction of a metal to a less toxic species, and direct efflux of a metal out of the cell. Finding novel species is of great interest in the face of potential bioremediation application. The objectives of this work are to aimed isolate and to characterize novel species of heavy metal-resistant and heavy metal-solubilizing bacteria from mine soils of Dabaoshan locating at south China.

## Materials and Methods

### Strain Isolation

Soil samples were collected from Dabaoshan Mine. 25 g soil samples were serially diluted with 225 mL 0.85% NaCl (w/v) and suitable 10-fold dilutions were plated onto MGY agar with Cd^2+^ (KCl 0.01%; MgS0_4_ **^.^**7H_2_0 0.025%; (NH_4_)_2_SO_4_ 0.2%; K_2_HP0_4_ 0.025%; Glucose 0.1%; Yeast extract 0.01%;1 mM Cd^2+^; Agar 2.0%) (Difco). The plates were incubated at 28°C for 4 days and all colonies were isolated. Among the isolation, a strain of purple color was isolated, designated as strain GIMN1.004^T^.

### Morphological and Physiological Characteristics

Gram reaction was determined according to the method described by Smibert & Krieg [9] after incubation at 28°C or 5 day on MGY agar. Cell morphology was observed by transmission electron microscopy (HITACHI H 7650) and phase-contrast microscopy (E600; Nikon) after incubation at 28°C for 4 day on MGY agar. Catalase activity was determined by assessing bubble production with 3% (v/v) H_2_O_2_, and oxidase activity was determined using 1% (w/v) tetramethyl-p-phenylenediamine after incubation at 28°C for 4 day on MGY agar. Growth after 5 days incubation in MGY liquid medium was assessed at different temperature (4, 18, 25, 30, 37 and 42°C) and various pH conditions (pH 4.0, 5.5, 6.0, 6.5, 7.0, 7.5, 8.0, 8.5, 9.0, 9.5 and 10.0), respectively. For the pH experiment, three different buffers were used (final concentration, 50 mM): acetate buffer for pH 4.0–5.5; phosphate buffer for pH 6.0–8.0; Tris buffer pH 8.5–10.0. Salt tolerance was tested in MGY supplemented with 1–10% (w/v) NaCl after 5 days of incubation at 28°C Anaerobic growth was assessed using incubation at 28°C for 5 days in 10 ml rubber-stoppered, screw-capped tubes containing MGY medium (9 ml) covered with liquid paraffin. Indole production and the Voges–Proskauer reaction were tested by using standard procedures[9] after incubation at 28°C for 5 day on MGY agar.

Other physiological characteristics and the utilization of various substrates as sole carbon sources were determined using the Biolog GN2 MicroPlates (Biolog Identification System) assay as recommended by the manufacturer. Sample preparation and analysis were performed according to the directions of the manufacturer (Biolog GN2 System). The triplicate microplates were read, after 4 h and 24 h incubation, using Microstation hardware (Biolog). The data were analyzed using MICROLOG 3 software (Biolog). All the experiments were carried out in triplicate.

### GC Content, PCR Amplification, Sequencing and Phylogenetic Analysis

Sequencing and assembly of 16S rRNA genes were carried out as described by Bakermans & Madsen [10]. The 16S rRNA gene was amplified using the universal primers 27F (5′-AGAGTTTGATCCTGGCTCAG-39) and 1492R (5′- GGTTACCTTGTTACGACTT-39) [11]. The amplified products were purified and sequenced using an automated capillary DNA sequencing system (ABI 3730) and a Bigdye Terminator cycle sequencing kit. The 16S rRNA gene sequence of strain GIMN1.004^T^ was compared with available 16S rRNA gene sequences from GenBank using the BLAST program (http://www.ncbi.nlm.nih.gov/blast/) to determine the approximate phylogenetic affiliation and was aligned with closely related members using CLUSTALW software [12].

A phylogenetic tree of *Burkholderia* 16S rRNA gene sequences was constructed using the neighbour-joining method of Saitou & Nei [13] with CLUSTAL W (version 1.81) and MEGA Version 3.1[14],. For the neighbour-joining analysis, a distance matrix was calculated according to Kimura’s two-parameter correction model. The Minimum Evolution and Maximum Parsimony methods were also used for tree construction, and the stability among the clades was assessed by employing 1000 replicate datasets. Sites with missing data were removed and the mitochondrial region sequences were used to test models of evolution. The p-distance model of evolution was used and employed to MP, ME and NJ tree, the nucleotide sequences and relations were analyzed MP, ME and NJ as implemented in MEGA 3.1. Branches marked with an asterisk are conserved in all methods used. This tree shows the close phylogenetic association of strain GIMN 1.004^T^ with certain members of other *Burkholeria* species.

To measure the G+C content of the chromosomal DNA, genomic DNA from the novel strain was extracted and purified as described by Moore & Dowhan [15], and the G+C content was determined as described by Mesbah *et al* [16], using reversed-phase HPLC. The experiment was carried out in triplicate.

### Chemotaxonomy Characteristics

Isoprenoid quinones were extracted from lyophilized cells which cultivated on MGY medium for 7 days at 28°Cwith chloroform/methanol (2∶1, v/v), evaporated under vacuum conditions and reextracted in n-hexane/water (1∶1, v/v). The crude n-hexane-quinone solution was purified using Sep-Pak Vac silica cartridges (Waters) and subsequently analyzed by HPLC (UltiMate 3000, Dionex) as described by Xie & Yokota [17]. Cellular fatty acids were determined for strains grown on NA at 30°C for 5 days. The fatty acid methyl esters were prepared according to the protocol of the Sherlock Microbial Identification System (MIDI system; http://www.midi-inc.com/) and analyzed by GC (6890; Hewlett Packard) using the Microbial Identification software package [18]. All the experiments were carried out in triplicate.

### DNA–DNA Hybridization

DNA–DNA hybridization was performed to evaluate the genomic DNA–DNA relatedness between strain GIMN1.004^T^ and *Burkholderia soli* GP25-8^T^ which was obtained from the KACC. DNA relatedness studies were conducted by using the fluorometric microdilution plate method [19]. All the experiments were carried out in triplicate.

### Minimum Inhibitory Concentration (MIC) of Heavy Metals and antibiotic resistance of the isolate

The MIC of the Cd^2+^ and Pb^2+^ for strain GIMN1.004^T^ and its closest phylogenetic neighbours *(B. soli* GP25-8^T^ and Burkholderia caryophylli ATCC 25418^T^) were determined by the plate dilution method as adopted by Summers and Silver [20] and Aleem *et al*. [21]. The lowest concentration that prevented bacterial growth was considered the MIC. The MIC was determined by the intersection of the relative survival curve with the horizontal axis. The relative survival curve was produced using weight changes of cultures that were supplemented with different concentrations of Cd^2+^ and Pb^2+^ compared with non-Cd^2+^ and Pb^2+^ -supplemented controls under the same conditions. The experiments were carried out in triplicate. Cultures were incubated on NA media at 30°C for 7 d.

The NA agar medium was used for the antibiotic resistance experiments. Stationary-phase broth cultures of GIMN1.004^T^ were used as inocula in the antibiotic resistance tests. Plates containing an antibiotic, as well as a nonselective control plate, were streaked with portions (approximately 10 µl) of inoculum, and growth was scored after 5 days. Resistance to a particular concentration of antibiotic was defined as the ability of a strain to form colonies at that concentration. Stock solutions of kanamycin (10 mg/L,20 mg/L), streptomycin (10 mg/L, 20 mg/L), ampicillin (100 mg/L, 200 mg/L) and cefetamet (35 mg/L,70 mg/L) were prepared in distilled water; All solutions were filter-sterilized using 0.45-µm membrane filters. Antibiotics were added to molten agar after sterilization and cooling to 50°C. The NA agar medium without antibiotics was used as controls. The experiments were carried out in triplicate. Cultures were incubated at 28°C for 7 d.

### Analysis of Cadmium Adsorbability

To determine the Cd^2+^ content of bacterial cells grown in NA supplemented with 185.56 mg/L CdCl_2_ for 48 h cells were harvested, rinsed three times using TSB/10 and dried at 55°C for 24 h. Following addition of 5 ml HNO_3_ (70% ), mineralization was carried out in a microwave oven. Metal content was determined using an inductively coupled plasma atomic emission spectrometry (ContrAA700). The experiments were carried out in triplicate.

Stain GIM1.004^T^ was incubated in MGY medium at 0, 2 or 8 mM Cd^2+^ at 150 r/min and 30°C for 24 h. After centrifugation at 12000 g for 10 min, precipitated cells were washed with deionized water for 3 times and then vacuum-dried. One mg dried samples were thoroughly mixed with 100 mg KBr and pressed into slice at 10 t/cm^2^ for 1 min. The slice was analyzed with FTIR spectroscopy [22]. According to the infrared spectrum of functional groups in cell wall changed under Cd stress, the mechanism of Cd absorption by cells was elucidated.

### Statistical Analysis

Analysis of variance and the Student–Newman–Keuls test (p<0.05) were used to compare treatment means. All the statistical analyses were carried out using SPSS 10.0.

## Results and Discussion

**Figure 1:**
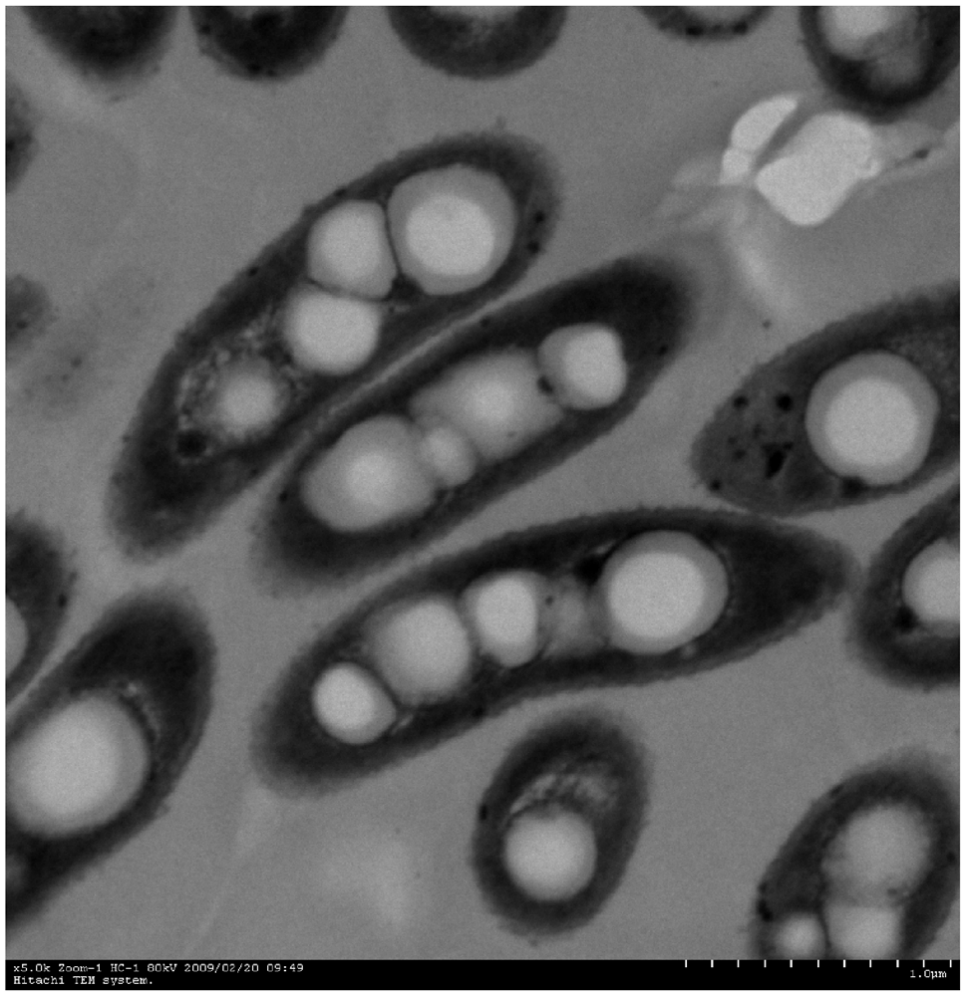
Transmission electron micrograph of a cell of strain GIMN1.004^T^, bar 1 µm.

Strain GIMN1.004^T^ was found to be Gram-negative, non-spore-forming, aerobic, weakly motile. In solid MGY agar medium (pH 4.0), the isolate formed round, light pink to white, opaque, wrinkled, and umbonate colonies with fuzzy boundaries. The cells of GIMN1.004^T^ were rod−shaped, 1.3−1.8 µm in length and 0.5−0.6 µm in diameter (Figure 1). In MGY agar medium with Cd^2+^ (8 mM), the isolate produced purple crystal particle. Strain GIMN1.004^T^ grew at pH 4.0–9.0 at 28°C, but optimally at pH 6.5. It grew at a temperature ranging from 5 to 40°C at pH 6.5, with an optimum growth at 30°C. It grew well at NaCl concentrations of 0–7% (w/v), but was slightly inhibited by NaCl concentration over 7%. The isolate was resistant to streptomycin (20 µg ml^−1^), kanamycin (20 µg ml^−1^), ampicillin (200 µg ml^−1^), and cefetamet (20 µg ml^−1^); The physiological and biochemical characteristics, metabolic properties and substrate-utilization results obtained for strain GIMN1.004^T^ were presented in Table 1.

**Table 1:** Physiological and biochemical characteristics that serve to differentiate strain GIMN1.004^T^ from its closest phylogenetic neighbours.

Characteristic	1	2	3
**Colony colour**	**Light pink**	**White**	**White**
Catalase	−	+	+
Motility	w	−	**+**
Utilization of:			
Adonitol	+	−	+
D-arabinose	+	−	+
D-arabitol	+	−	+
L-fucose	V	+	+
Maltose	+	−	−
D-raffinose	+	−	+
L-rhamnose	V	−	+
D-sorbitol	+	−	+
Sucrose	+	−	+
D-trehalose	−	−	+
Xylitol	+	−	+
α-ketovalevic acid	V	−	−
Malonic acid	−	−	**+**
Chemotaxonomy			
DNA G+C content (mol%)	61.6	64.9	65.3

Strains: 1, strain GIMN1.004^T^; 2, *Burkholderia soli* GP25-8^T^ [30]; 3, *Burkholderia caryophylli* ATCC 25418^T^ [31];

+, Positive; –, negative; V, variable reaction; W, weak reaction; ND, no data available.

All three strains could use D-fructose as sole carbon sources, and couldn’t use D-cellobiose. Lactose and D-melibiose.

The isolate was positive for utilization of Tween 40 and 80,*N*-acetyl-D-glucosamine, adonitol,L-arabinose, D-arabitol,D-fructose, L-fructose, D-galactose, α-D-glucos,m-inositol,D-mannitol, D-mannose,D-raffinose, D-sorbitol,sucrose, maltose, xylitol, pyruvic acid methylester,succinic acid mono methylester, *cis-*aconitic acid, formic acid, D-gluconic acid, α-hydroxybutyric acid, β-hydroxybutyric acid, *p*-hydroxy phenylacetic acid, α-keto butyric acid, D,L-lactic acid, propionic acid, quinic acid, D-glucosaminic acid, sebacic acid, succinic acid, bromosuccinic acid, succinamic acid, L-alaninamide,L-alanine,L-alanylglycine, L-asparagines, L-aspartic acid, L-glutamic acid, glycyl-L-glutamic acid, L-histidine, L-phenylalanine, L-proline, L- pyroglutamic acid, L-serine, L-threonine, γ-amino butyric acid, 2- aminoethanol,glycerol, D-glucose-6-phosphate as sole carbon sources. it was negative for the utilization of α-cyclodextrin, *N*-acetyl-D-galactosamine, i-erythritol, gentiobiose, D-melibiose, β-methyl-D-glucoside, D-trehalose, D-cellobiose, turanose, γ- -hydroxybutyric acid,α- ketoglutaric acid,malonic acid, glucuronamide, glycyl-L-aspartic acid, D-serine, D,L-carnitine, inosine, thymidine, phenyethylamine, putrescine, α-D-glucose-1-phosphate (Biolog GN2 Microplate system).

A 1437-bp 16S rRNA gene sequence was determined for strain GIMN1.004^T^. A BLAST search [23] of the GenBank database using this sequence showed high similarity to that of *Burkholderia*. The 16S rRNA gene sequence of strain GIMN1.004^T^ showed a similarity level of 99.4% (over 1428 bases) to that of *Burkholderia soli* strain GP25-8^T^ (GenBank accession No. DQ465451), and of 96–98% to that of other *Burkholderia* species and Burkholderiaceae bacteria. Comparison of the culture characteristics of strain GIMN1.004^T^ and its closest phylogenetic neighbours, however, revealed significant differences from *Burkholderia soli* GP25-8^T^ (Table 1).

**Figure 2:**
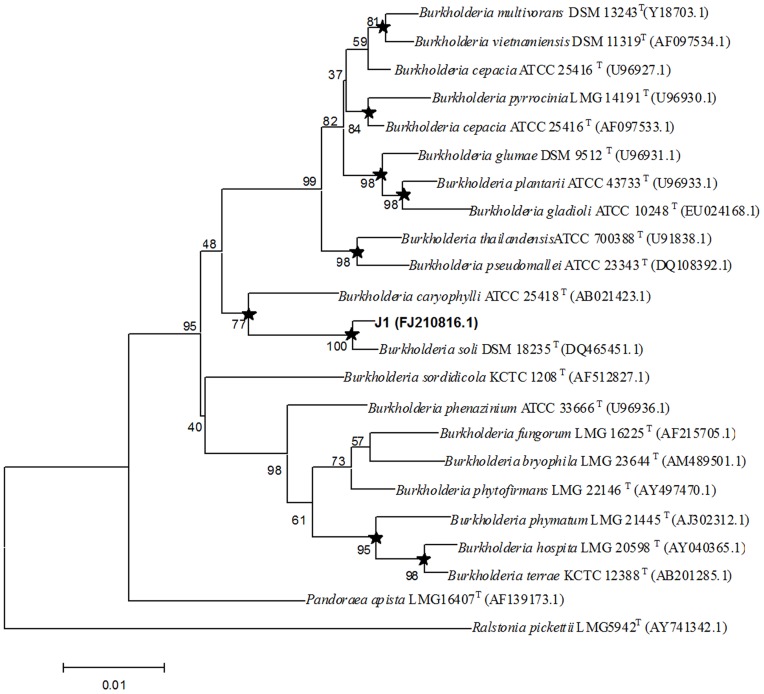
Unrooted Neighbour-joining tree constructed from *Burkholderia* 16S rRNA gene sequences, showing the phylogenetic relationship between *Burkholderia dabaoshanensis* sp. nov. and other *Burkholeria* spp. *Pandoraea apista* LMG16407^T^ and *Ralstonia pickettii* LMG5942^T^ was used as the outgroup. Bootstrap values (expressed as percentages of 1000 replications) greater than 50% are given at the nodes. GenBank sequence accession numbers are given in parentheses. Bar,0.01 substitutions per nucleotide position. The Minimum Evolution and Maximum Parsimony methods were also used for tree construction. Branches marked with an asterisk are conserved in all methods used.

In the phylogenetic tree (Figure 2), strain GIMN1.004^T^ clustered within the genus *Burkholderia*. Phylogenetic analysis of 16S rRNA gene sequences showed that strain GIMN1.004^T^ formed a cluster with *Burkholderia soli* GP25-8^T^, within the genus *Burkholderia*. Though the highest level of 16S rRNA gene sequence similarity (99.4%) was found with respect to *Burkholderia soli* DSM 18235^T^, the corresponding level of DNA–DNA relatedness as determined by hybridization was 25.0%. The values were well below the 70% cut-off point for species classification, as recommended by [24], thus confirming that the isolated strain is an independent novel species of the genus *Burkholderia*.

The chemotaxonomic characteristics of strain GIMN1.004^T^ were in agreement with the results of the phylogenetic inference, confirming that the novel bacterium belongs to the genus *Burkholderia*. The novel bacterium clearly differed from the related species in terms of cell-wall composition. The major quinones of GIMN1.004^T^ were ubiquinone Q-8, as in the case of other species of the genus *Burkholeria* [25]–[28]. Major cellular fatty acids (representing >5% of the total) were C_16∶0_ (25.05%), summed feature 8 (17.76% C_18∶1_ *ω7c* and C_18∶1_ *ω6c*), C_18∶0_ (10.57%), summed feature 3(5.93%, C_16∶1_ *ω7c* or iso-C_15∶0_ 2-OH ), C_17∶0_ cyclo (5.52%), C_18∶1_ *ω9c* (5.23%). while that of *Burkholderia soli* was C_18∶1_ *ω7c* (33.5%), summed feature 3 (23.8%, C_16∶1_ *ω7c* or iso-C_15∶0_ 2-OH), C_16∶0_ (15.3%), summed feature 2 (6.6%, C_14∶0_ 3-OH or C_16∶1_ iso I) (Table 2). The DNA G+C content of strain GIMN1.002^T^was 61.6 mol%.

**Table 2:** Cellular fatty acids composition (>1%) of GIMN1.004^T^ and its closest phylogenetic neighbours.

	*Burkholderia dabaoshanensis* GIMN1.004^T^	*Burkholderia soli* GP25-8^T^	*Burkholderia caryophylli* ATCC 25418^T^
C_12∶ 0_	1.77	1.8	−
C_13∶1_ AT 12–13	0.91	1.0	1.0
C_13∶0_ iso 3-OH	2.40	−	−
C_14∶0_	4.18	−	4.8
C_15∶1_ iso F	2.14	−	−
C_16∶0_	25.05	15.3	18.3
C_16∶0_ 2-OH	0.90	1.7	2.4
C_16∶0_ 3-OH	1.76	4.2	4.3
C_16∶1_ 2-OH	0.90	4.8	2.6
C_17∶0_ cyclo	5.52	1.0	2.1
C_18∶0_	10.57	−	−
C_18∶1_ 2-OH	0.73	2.7	3.2
C_18∶1_ *ω7c*	−	33.5	36.8
C_18∶1_ *ω9c*	5.23	−	−
C_19∶0_ cyclo *ω8c*	4.55	−	1.0
C_20∶4_ *ω6.9.12.15c*	2.35	−	−
Summed feature 1*	1.34	−	−
Summed feature 2*	2.74	6.6	6.1
Summed feature 3*	5.93	23.8	16.1
Summed feature 8*	17.76	−	−

* Summed feature 1 comprises C_15∶1_ iso H or C_13∶0_ 3-OH.

Summed feature 2 comprises C_14∶0_ 3-OH or C_16∶1_ iso I, or both;

Summed feature 3 comprises C_16∶1_ *ω7c* or C_16∶1_ *ω6c*, or both;

Summed feature 8 comprises C_18∶1_ *ω7c* and C_18∶1_ *ω6c.*

Data of *B.soil* and *B. caryophylli* come from Yoo et al. [30].

Strain GIMN1.004^T^ grew well in NA agar medium supplemented with ≤18 mM Cd^2+^ and visible growth was observed in the presence of 19–22 mM Cd^2+^. The MIC of Cd^2+^ for strain GIMN1.004^T^ was 22 mM. Strain GIMN1.004^T^ grew well in NA agar medium supplemented with ≤4 mM Pb^2+^ and visible growth was observed in the presence of 5–6 mM Pb^2+^. The MIC of Pb^2+^ for strain GIMN1.004^T^ was 6 mM. *B. soli* GP25-8^T^ and *B. caryophylli* ATCC 25418^T^ couldn’t grow in NA agar medium supplemented with ≤1 mM Cd^2+^ and Pb^2+^ under the same conditions. The testing of antibiotic resistance showed that stain can grow well at Amp, Kn, Cat and Str.

The testing results showed that the adsorbability of GIM1.004^T^ was 144.94 mg/g by atomic absorption, while the adsorbability of another 8 strain cadmium-tolerant bacteria which isolated under the same condition was 60 mg/g. The adsorptive mechanism of GIMN1.004^T^ for cadmium depended on the action of the amide,carboxy and phosphate of cell surface and producing low-molecular-weight (LMW ) organic acids to complex or chelated Cd^2+.^

Cadmium is an ubiquitous toxic metal that was capable of modulating immune responses [29]. In this study, we isolated one bacterial isolates which could be resistant to Cd^2+^. Based on the tolerant ability and higher adsorbability of the Cd^2+^, bacterial strain GIM1.004^T^ have potential implications cleaning up of detoxifying metal-contaminated soils in the future.

Therefore, based on the physiological, biochemical, chemotaxonomic, and molecular genetic results described above, GIMN1.004^T^ represented a new species of the genus *Burkholderia*, which has been tentatively named as *Burkholdria dabaoshanensis* sp. nov. (da.bao.shan.en'sis. N.L. fem. adj. dabaoshanensis pertaining to dabaoshan which the strain was isolated ) with the strain type GIMN1.004^T^ ( =  CCTCC 209109^T^ =  NRRL B-59553^T^ ).

## References

[1] Dudka S, Adriano DC (1997) Environmental impacts of metal ore mining and processing: A review. Journal of Environmental Quality 26: 590–602.

[2] Navarro MC, Perez-Sirvent C, Martinez-Sanchez MJ, Vidal J, Tovar PJ, et al (2008) Abandoned mine sites as a source of contamination by heavy metals: A case study in a semi-arid zone. Journal of Geochemical Exploration 96: 183–193.

[3] Wcisło E, Ioven D, Kucharski R, Szdzuj J (2002) Human health risk assessment case study an abandoned metal smelter site in Poland. Chemosphere 47: 507–515. 11996126 10.1016/s0045-6535(01)00301-0

[4] Kachenko AG, Singh B (2006) Heavy metals contamination in vegetables grown in urban and metal smelter contaminated sites in Australia. Water, Air, and Soil Pollution 169: 101–123.

[5] Siloniz M, Balsolobre C, Valderrama M, Peinado J (2002) Feasibility of copper uptake by the yeast *Pichia guilliermondii* isolated form sewage sludge. Res Microbiol 153: 173–180. 12002567 10.1016/s0923-2508(02)01303-7

[6] Kratochvil D, Volesky B (1998) Advances in the biosorption of heavy metals. Trends in Biotechnology 16 (7): 291–300.

[7] Coenye T, Vandamme P (2003) Diversity and significance of Burkholderia species occupying diverse ecological niches. Environ Microbiol 5: 719–729. 12919407 10.1046/j.1462-2920.2003.00471.x

[8] O’Sullivan LA, Mahenthiralingam E (2005) Biotechnological potential within the genus Burkholderia. Lett Appl Microbiol 41: 8–11. 15960745 10.1111/j.1472-765X.2005.01758.x

[9] Smibert RM, Krieg NR (1994) Phenotypic characterization. In Methods for General and Molecular Bacteriology, 607–654p. Gerhart P, Murray RGE, Wood WA, Krieg NR, editors. Washington DC: American Society for Microbiology.

[10] Bakermans C, Hohnstock-Ashe AM, Padmanabhan S, Padmanabhan P, Madsen EL (2002) Geochemical and physiological evidence for mixed aerobic and anaerobic field biodegradation of coal tar waste by subsurface microbial communities. Microb Ecol 44: 107–117. 12087424 10.1007/s00248-002-3011-y

[11] Lane DJ (1991) 16S/23S rRNA sequencing. In Nucleic Acid Techniques in Bacterial Systematics 115–147p. Edited by Stackebrandt E, Goodfellow M. New York: Wiley.

[12] Thompson JD, Gibson TJ, Plewniak F, Jeanmougin F, Higgins DG (1997) The CLUSTAL_X windows interface: flexible strategies for multiple sequence alignment aided by quality analysis tools. Nucleic Acids Res 25: 4876–4882. 9396791 10.1093/nar/25.24.4876 PMC147148

[13] Saitou N, Nei M (1987) The neighbor-joining method: a new method for reconstructing phylogenetic trees. Mol Biol Evol 4: 406–425. 3447015 10.1093/oxfordjournals.molbev.a040454

[14] Kumar S, Tamura K, Nei M (2004) MEGA3: integrated software for molecular evolutionary genetics analysis and sequence alignment. Brief Bioinform 5: 150–163. 15260895 10.1093/bib/5.2.150

[15] Moore DD, Dowhan D (1995) Preparation and analysis of DNA. In Current Protocols in Molecular Biology, 2–11p. Edited by Ausubel FW, Brent RE.

[16] Mesbah M, Premachandran U, Whitman WB (1989) Precise measurement of the G+C content of deoxyribonucleic acid by highperformance liquid chromatography. Int J Syst Bacteriol 39: 159–167.

[17] Xie CH, Yokota A (2003) Phylogenetic analyses of Lampropedia hyalina based on the 16S rRNA gene sequence. J Gen Appl Microbiol 49: 345–349. 14747976 10.2323/jgam.49.345

[18] Sasser M (1990) Identification of bacteria by gas chromatography of cellular fatty acids, MIDI Technical Note 101. Newark, DE: MIDI Inc.

[19] Ezaki T, Hashimoto Y, Takeuchi N, Yamamoto H, Liu SL, et al (1988) Simple genetic method to identify viridans group streptococci by colorimetric dot hybridization and fluorometric hybridization in microdilution wells. J Clin Microbiol 26: 1708–1713. 3183018 10.1128/jcm.26.9.1708-1713.1988 PMC266701

[20] Summers AO, Silver S (1972) Mercury resistance in a plasmid bearing strains of Escherichia coli. J Bacteriol 112: 1228–1236. 4565536 10.1128/jb.112.3.1228-1236.1972 PMC251553

[21] Aleem A, Isar J, Malik A (2003) Impact of long-term application of industrial wastewater on the emergence of resistance traits in Azotobacter chroococcum isolated from rhizosphere soil. Bioresour Technol 86: 7–13. 12421001 10.1016/s0960-8524(02)00134-7

[22] Kamnev AA, Antonyuk LP, Matora LY (1999) Spectroscopic characterization of cell membranes and their constituents of the plant-associated soil bacterium *Azospirillum brasilense* [J]. Molecular structure 480–481: 387–393.

[23] Altschul SF, Madden TL, Schaffer AA, Zhang J, Zhang Z, et al (1997) Gapped BLAST and PSI-BLAST: A new generation of protein database search programs. Nucl. Acids Res 25: 3389–3402. 10.1093/nar/25.17.3389 PMC146917 9254694

[24] Wayne LG, Brenner DJ, Colwell RR, Grimont PAD, Kandler O, et al (1987) Report of the ad hoc committee on reconciliation of approaches to bacterial systematics. Int J Syst Bacteriol 37: 463–464.

[25] Yamada Y, Takinami-Nakamura H, Tahara Y, Oyaizu H, Komagata K (1982) The ubiquinone systems in the strains of *Pseudomonas* species. J Gen Appl Microbiol 28: 7–12.

[26] Zhang H, Hanada S, Shigematsu T, Shibuya K, Kamagata Y, et al (2000) *Burkholderia kururiensis* sp. nov., a trichloroethylene (TCE)-degrading bacterium isolated from an aquifer polluted with TCE. Int J Syst Evol Microbiol 50: 743–749. 10758884 10.1099/00207713-50-2-743

[27] Yang HC, Im WT, Kim KK, An DS, Lee ST (2006) *Burkholderia terrae* sp. nov., isolated from a forest soil. Int J Syst Evol Microbiol 56: 453–457. 16449457 10.1099/ijs.0.63968-0

[28] Valverde A, Delvasto P, Peix A, Velázquez E, Santa-Regina I, et al (2006) *Burkholderia ferrariae* sp. nov., isolated from an iron ore in Brazil. Int J Syst Evol Microbiol 56: 2421–2425. 17012573 10.1099/ijs.0.64498-0

[29] Fujimaki H, Ishido M, Nohara K (2000) Induction of apoptosis in mouse thymocytes by cadmium. Toxicology Letters 115: 99–105. 10802385 10.1016/s0378-4274(00)00178-8

[30] Yoo SH, Kim BY, Weon HY, Kwon SW, Go SJ, et al (2007) *Burkholderia soli* sp. nov., isolated from soil cultivated with Korean ginseng. Int J Syst Bacteriol 57: 122–125. 10.1099/ijs.0.64471-0 17220453

[31] Bramer CO, Vandamme P, Silva LF, Gomez JGC, Steinbuchel A (2001) *Burkholderia sacchari* sp. nov., a polyhydroxyalkanoate-accumulating bacterium isolated from soil of a sugar-cane plantation in Brazil. Int J Syst Bacteriol 51: 1709–1713. 10.1099/00207713-51-5-1709 11594600

